# Laboratory Investigation of the Strength Degradation against Ultraviolet Radiation of Geonets for Slope Protection

**DOI:** 10.3390/ma17194803

**Published:** 2024-09-29

**Authors:** Rui Zhang, Huan Wang, Zhengnan Liu, Xiang Wang, Heping Yang, Yani Zhang

**Affiliations:** 1 School of Traffic and Transportation Engineering, Changsha University of Science & Technology, Changsha 410114, China; zr@csust.edu.cn (R.Z.); whhh@stu.csust.edu.cn (H.W.); yhp@stu.csust.edu.cn (H.Y.); zyn_csust@163.com (Y.Z.); 2 National Key Laboratory of Green and Long-Life Road Engineering in Extreme Environment (Changsha), Changsha University of Science & Technology, Changsha 410114, China; 3 Engineering Research Center of Catastrophic Prophylaxis and Treatment of Road & Traffic Safety of Ministry of Education, Changsha University of Science & Technology, Changsha 410114, China; 4 Hunan Communications Research Institute Co., Ltd., Changsha 410015, China; 5 China Railway Siyuan Survey and Design Group Co., Ltd., Wuhan 430063, China; wangxiangzt@126.com

**Keywords:** road engineering, strength degradation, tension test, slope protection, geonet

## Abstract

Sufficient light and high UV intensity pose significant challenges to the long-term performance of polymeric geonet materials for slope-protection structures. This study investigates strength degradation under the effect of UV radiation; five different types of geonets were selected, which can be categorized as polyamide (PA), polypropylene (PP), and polyethylene (PE) materials. A comprehensive experimental investigation was performed, including tension strength, peer strength, artificially accelerated aging, and SEM tests, to further establish a service life prediction method used for slope-protection design. The results showed that the tension strength, percentage of breaking elongation, and peer strength all depict a descending trend with aging-elapsed time, especially in the early 600 h. The decreasing tendency of these mechanical properties’ magnitude differed in the diversity of direction and material type. Significant changes have been generated on the geonet surface after aging; materials with smooth surfaces exhibit a strong ability against strength degradation. Fitting results affirmed the predictive technique as a useful engineering tool for tension strength assessments, offering guidelines for using and designing of geonets for slope-protection structures.

## 1. Introduction

The use of polymeric geonet materials in constructing slope-protection structures, such as geogrids or geotextiles, has witnessed a remarkable surge in recent decades. The preference for these materials over more conventional ones stems from many advantages, encompassing ease of use and installation, cost-effectiveness, and seamless integration with the natural environment. These materials are capable of executing a diverse array of functions, including filtration, drainage, separation, protection, and reinforcement, making them ideally suited for application in a wide spectrum of civil engineering infrastructures, such as landfills, roads, railways, reservoirs, tunnels, and coastal protection structures. However, they should encounter various agents that can adversely affect their performance both immediately and over an extended period in their application as slope-protection structures [1]. In the southern region of China, sufficient light (1200 h~3000 h per year) and high UV intensity (3500 MJ/m^2^~5850 MJ/m^2^ per year) pose significant challenges to the long-term performance of the polymeric materials for slope-protection structures, which were, after all, designed for a service life of more than 20 years in many cases. Therefore, investigation of their strength degradation against ultraviolet radiation and its mechanism should be necessary to facilitate subsequent design and ensure the structures’ long-term performance.

As slope-protection structures ensure the safety of engineering practices and the sustainability of the environment, these types of construction elements have received extensive attention, leading to the development of various innovative solutions. Vegetation plays a crucial role in slope protection. Common technologies used in slope vegetation restoration include soil spraying [2], vegetation concrete slope protection [3], thick base material application [4], and hydraulic spraying [5]. These techniques include soil and water conservation, air purification, and landscape restoration [6]. However, the strength of vegetation is relatively low, which makes it difficult to effectively resist severe geologic hazards, such as landslides and mudslides [7]. Moreover, its scope of application is limited by the slope gradient, geologic conditions, and moisture status. In addition, it is also limited in its ability to hold soil and water, especially under rainfall, which tends to form runoff gullies and erosion [8]. Flexible structures such as concrete canvas, geogrid, and soil-bag have also been widely used in slope protection [9,10,11,12]. Furthermore, it shows its advantages in rapid construction, low labor costs, and quick strength development [13,14]. Compared to vegetation, these types of engineering elements enhance the overall stability and strength of slopes. Nevertheless, the mechanical behavior of geosynthetic materials within these structures would degrade during service life because of the continuous environmental changes outside the slope; the stability and durability would also face significant challenges.

Geonets combined with vegetation effectively stabilize vegetation and soil, prevent landslides and erosion, and provide high construction efficiency and low cost [15]. With increasing demands for ecological sustainability, geonets are widely adopted as slope-protection structures [16] and have proven long-term reliability [17]. Recently, geonets have been extensively explored in research through both numerical simulations and experimental methods, focusing on their mechanical behavior [18,19,20], providing insights into their tensile strength and modulus variations. These studies collectively contribute to the understanding and optimizing of geosynthetics for engineering applications, underscoring the importance of material properties and testing methods in enhancing geosynthetic performance. Additionally, factors such as ultraviolet radiation, temperature, and oxidation significantly impact the materials’ performance, and the materials’ response to the environment also aroused attention. Terahertz dielectric spectroscopy [21], ATR-IR spectroscopy [22], and scanning electron microscopy [23] were utilized to study the effects of ultraviolet radiation, discovering that the depletion of stabilizers leads to oxidative degradation [24]. The microstructural changes occurred during tensile failure [25]. Koerner proposed models predicting lifespan under covered and uncovered conditions, highlighting the crucial role of antioxidants in material durability [26]. Fewer studies were concerned with predicting the mechanical behavior of materials during aging under ultraviolet radiation in service life. However, researchers should develop more precise simulation techniques and standardized testing methods to improve the reliability of performance predictions.

The aim of this study was to investigate the effects of UV radiation on the mechanical behavior of commonly used polymeric materials for slope protection in China to propose a prediction method for the materials in real working conditions. A comprehensive set of tests was performed, including mechanical tests before and after aging by accelerated aging in the laboratory and microstructural observation through scanning electron microscopy. Subsequently, the evolution of tensile strength, percentage of breaking elongation, and peel strength of these materials after different aging-elapsed times were investigated, and differences in the parameters among these materials were compared and discussed based on the SEM photos. Furthermore, a prediction method of tension strength of the materials was proposed for selecting the polymeric materials in slope-protection-structure design through environmental variables (local solar radiation and light duration) and the material’s mechanical properties (initial tension strength).

## 2. Materials and Methods

### 2.1. Slope Protection Material

**Figure 1:**
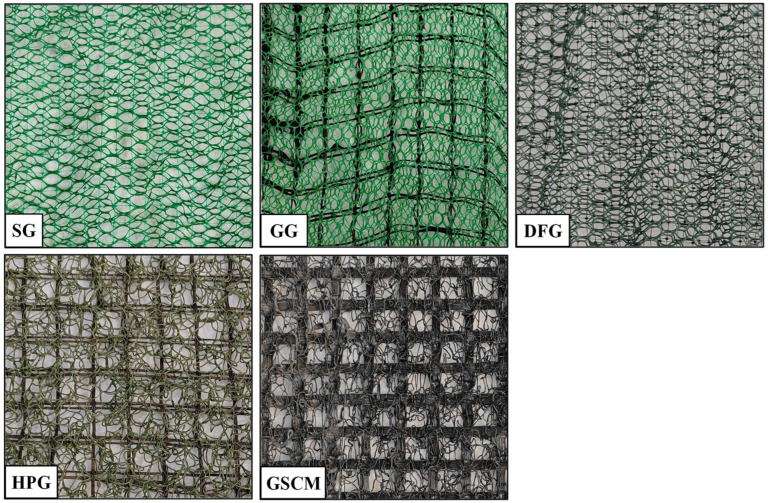
Slope-protection materials.

Five different types of common three-dimensional (3D) geonets in China were used for a laboratory investigation of the aging characteristics, including standard geonet (SG), gridded geonet (GG), double-foamed geonet (DFG), high-performance geonet (HPG), and gridded soil conservation mat (GSCM). The GSCM material is made of polyamide (PA), the HPG is made of polypropylene (PP), and the remaining geonets are made of polyethylene (PE). All the geonets have a particular strength and elasticity and can be used for slope-surface protection with plants from erosion by rainfall. While their exterior structure and mechanical properties are diverse, the appearance and structure of these materials are shown in Figure 1, and the basic properties are depicted in Table 1.

### 2.2. Test Procedure and Methods

**Figure 2:**
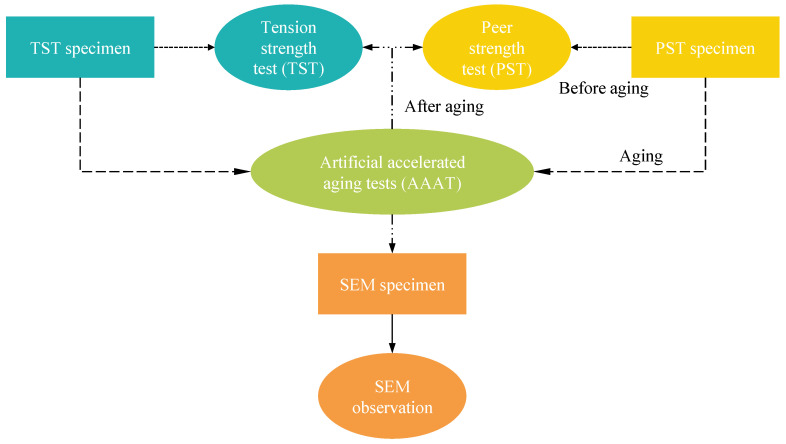
Flowchart of the testing procedure.

In this study, a series of laboratory tests were designed to investigate the physical properties of geonet materials during the aging process and to further establish a service life prediction method used for slope protection design. The testing procedure is depicted in Figure 2. After specimen preparation, most specimens were placed in accelerated test devices for aging; the rest of the specimens were used for the tension strength and peer strength tests before aging to obtain their initial strengths. Subsequently, the specimen underwent different aging-elapsed times and was taken out of the aging device for further strength test and SEM observation. More information will be provided in the following sections.

#### 2.2.1. Specimen Preparation

During specimen preparation, for each type of 3D geonet material, the specimens for the tension strength test and artificially accelerated aging test were cut randomly along longitudinal and transverse directions, respectively, from the origin materials. These specimens were square in shape with a side length of 200 mm. As for the peer strength test, the specimen underwent different aging times and was further cut into 200 mm × 50 mm, according to the *Chinese specification for test and measurement of geosynthetics* (SL 235) [27].

In total, 240 specimens were prepared for subsequent testing, 48 specimens for each geonet material. As for each tension strength or peer strength testing at different aging-elapsed time, 3 specimens were used for parallel testing.

#### 2.2.2. Artificial Accelerated Aging Test

**Figure 3:**
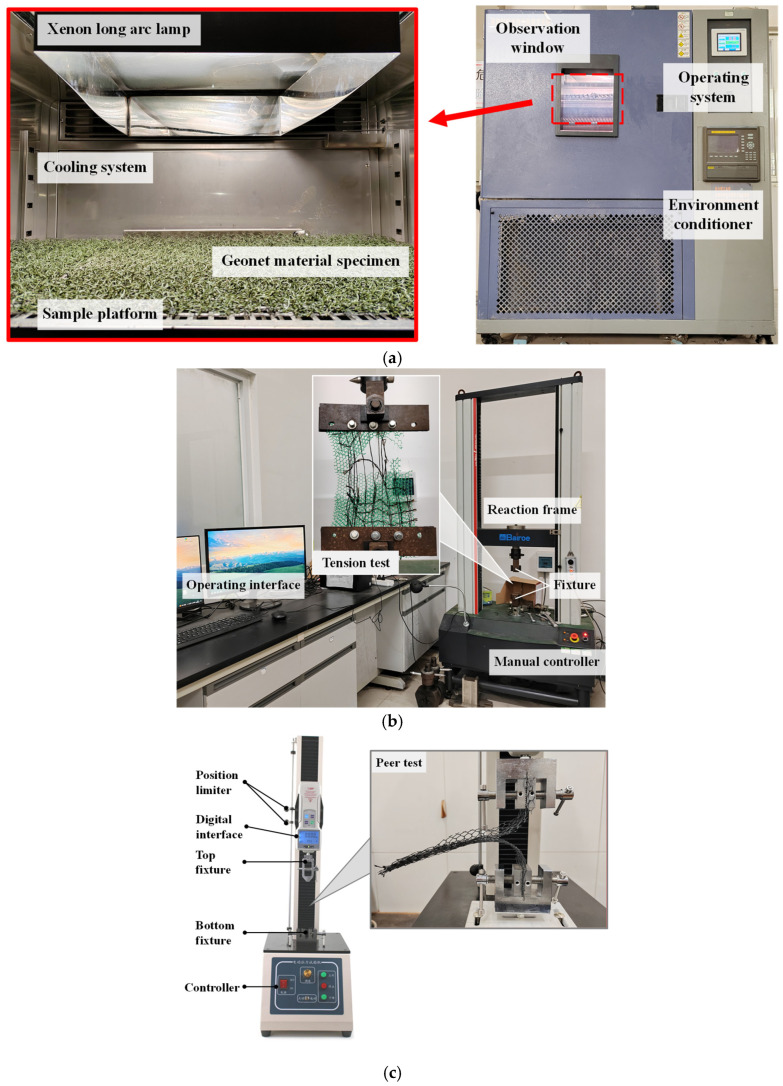
The testing devices used in this study: (**a**) the SX432S Xenon lamp weather resistant aging tester for AAAT; (**b**) the microcomputer-type electronic universal testing machine WDW-50 for tension test; (**c**) the AXL-S-400 electric tensile testing machine for peer strength test.

The laboratory artificial accelerated aging test (AAAT) is designed to investigate 3D geonets’ aging behavior in a short period of time, which artificially enhances or simulates the actual working conditions of the climatic environment. To effectively simulate the effects of various factors, such as light, high temperature, rain, humidity, and condensation under natural conditions, the SX432S Xenon Lamp Weather Resistant Aging Tester (Figure 3a) manufactured by SPECTRO Environmental Instruments Co., Ltd. (Guangzhou, China) was selected and used in this study, which can meet the requirement of *Standard Practice for Exposing Nonmetallic Materials in Accelerated Test Devices that Use Laboratory Light Sources* (ASTM G151-10) [28]. The spectral range of this device varies from 290 nm to 800 nm, and the irradiation intensity can be controlled and continuously adjusted from 20 W/m^2^ to 1200 W/m^2^. The temperature and the humidity can be performed from −40 °C to 150 °C and 20% to 98%, respectively.

This equipment uses the xenon long arc lamp as an ultraviolet ray (UV) source to simulate enhanced sunlight, and the moisture generated by the cooling system to simulate natural rain. Thus, it forms a specific temperature, humidity, and sunlight environment. The material is subjected to show its aging behavior in a short period of time in this reproduced simulated long-term natural environment.

In the pre-study, the authors found it hard to distinguish the diversity of material performance by completely following the testing condition in the specification. Thus, the environment the specimens underwent aging in was modified. After specimen preparation, each geonet specimen group was placed on the equipment sample platform. It was set up as a 12 h cycle of alternating light (11 h) and condensation (1 h, including 5 min for water spray). The geonets, when used in applications such as soil reinforcement, are often exposed to a 70% humidity condition. And the humidity is essential to simulate the hydrophilic degradation mechanisms that could occur over time, such as hydrolysis or changes in mechanical properties due to moisture absorption [29]. Thus, to approximate a real-world environmental condition, the humidity was set constant in the cycle at 70% during the test. The temperature of the black panel was set at 70 °C, and the irradiation intensity was 600 W/m^2^ in the light procedure. Meanwhile, in condensation, the temperature was set at 25 °C, and the irradiation intensity was set as 0 W/m^2^.

The aging program set for each testing series of materials was the same, which would last for 100 times of 12 h cycles (1200 h) in total and stop by different times for further mechanical testing. The specific timing in this study was set at 10, 20, 30, 40, 60, 80, and 100 times of a 12 h cycle, corresponding to the aging time of 120 h, 240 h, 360 h, 480 h, 720 h, 960 h, and 1200 h, respectively.

#### 2.2.3. Tension Strength Test

For further evaluation of aging characteristics, the tension test compares materials’ tensile and peer strengths before and after the aging process. The microcomputer-type electronic universal testing machine WDW-50 (Shanghai Bairoe Test Instrument Co., Ltd., Shanghai, China). (Figure 3b) was used in this study. The WDW-50 microcomputer-controlled electronic universal testing machine is a powerful testing equipment with key technical parameters showcasing its high precision, wide range, and flexibility. It boasts a maximum testing force of 50 kN, catering to high-strength testing requirements of various materials. Its testing force measurement range spans from 2% to 100% of full scale, with a measurement accuracy superior to ±1% of the indicated value, ensuring the accuracy of test results. In terms of displacement measurement, the machine achieves a high resolution of 0.001 mm and an accuracy of ±1%, capable of precisely capturing minute deformations of samples. Furthermore, the WDW-50 offers a wide speed range, with the crosshead speed adjustable from 0.001 to 500 mm/min without steps, satisfying the speed requirements of different materials under various testing conditions. With an effective testing width of 400 mm and tensile/compression strokes of up to 650 mm/800 mm (depending on the model), it provides ample testing space for samples of various sizes.

During the test for tensile strength, according to the *Test Methods of Geosynthetics for Highway Engineering JTG E50* (2006) [30], the requirements for the tensile rate of different types of three-dimensional geonets were referenced. The tensile rate set in the test was 200 mm/min, and it was ensured that the direction of the measured properties of the material was kept parallel to the direction of the applied load. The clamping length of the specimen was set at 100 mm. The peak value of tensile force was defined as the breaking tensile force of the material, and the test was stopped when all layers of the material had broken. Moreover, to ensure the consistency of the test conditions, after preparation, all specimens were placed at room temperature of (20 ± 2) °C for at least 24 h before testing and then tested under the same environmental conditions.

#### 2.2.4. Peer Strength Test

As for testing the peer strength of materials, the AXL-S-400 electric tensile testing machine (Deep Measurement Intelligence (Shenzhen) Co., Ltd., China) (Figure 3c) was used. This electric tensile testing machine boasts a wide testing range from 0 to 1000 N (or equivalently 100 kgf), ensuring high versatility. With a satisfactory resolution of up to 1 N (or 0.1 kgf), it precisely captures subtle changes during testing. The machine achieves a high accuracy standard of ±0.5%, guaranteeing accurate and reliable test results. Additionally, it features a 400 mm testing stroke, suitable for testing samples of various lengths. The adjustable test speed, ranging from 30 to 300 mm/min, meets the velocity control requirements for different testing needs. The testing procedure followed the *Testing Procedure for Geosynthetics SL 235* (2012) [31]; the specimen experienced different aging times and was cut into pieces with 200 mm × 50 mm sizes. During testing, the tensile speed was controlled at 300 mm/min, and the peel length of the specimen should not be less than 100 mm.

#### 2.2.5. SEM Observation

Scanning electron microscopy (SEM) was performed with geonet specimens of 5 × 5 mm randomly removed from the samples before and after aging of 1200 h. A ZEISS SIGMA microscope (Carl Zeiss AG, Oberkochen, Germany), furnished with a secondary electron detector and operating at an electron acceleration voltage of 3.0 kV, was utilized. Each specimen was securely attached to the sample holder using a silver suspension and subsequently coated with a gold deposit to guarantee optimal electrical conductivity.

## 3. Results and Analysis

### 3.1. Aging Effect on Physical Properties

#### 3.1.1. Variation in Tension Strength

**Figure 4:**
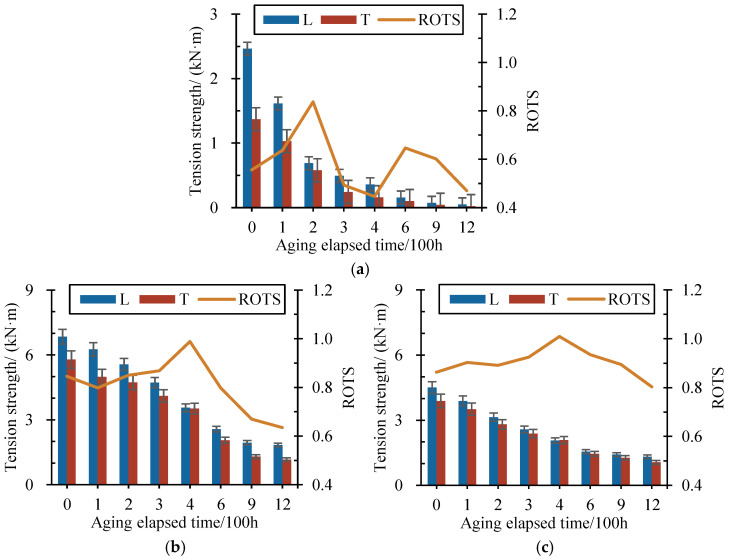

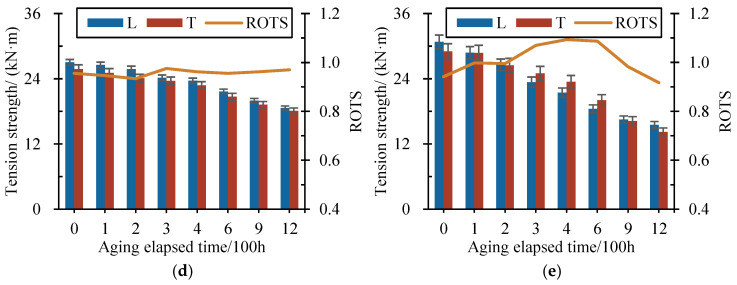
Variation in longitudinal (L) and transverse (T) tension strength and ratio of orthogonal tension strength (ROTS) with aging-elapsed time. (**a**) SG. (**b**) GG. (**c**) DFG. (**d**) HPG. (**e**) GSCM.

After tension strength tests and artificial accelerated aging tests, the tension strengths of each type of geonet after different aging-elapsed times were obtained. Figure 4 depicts the variation in longitudinal (L) and transverse (T) tension strength of each type of the 3D geonet with aging-elapsed time, where the ratios of orthogonal tension strength (ROTS) are also demonstrated, calculated by dividing the transverse tension strengths by longitudinal ones.

As shown in Figure 4, the tension strength among different geonet materials and directions all showed a descending trend with aging-elapsed time, where the magnitude of the strength reduction was diverse. The SG had the smallest initial tension strength and almost lost its strength after 900 h of aging, followed up by the GG and the DFG, which had 2–3 times higher initial tensile strength than that of SG, while it also fell into the range of 1–2 kN/m of the residual strength after 1200 h of aging. The initial tensile strength of the HPG and the GSCM was one order of magnitude higher than that of other types of geonet materials. Comparing Figure 4d with Figure 4e, it can be observed that the decrease in the tension strength of the GSCM during the aging process was faster than that of the HPG, and the residual strength of the GSCM was lower despite the higher initial tensile strength. In addition, except for the strengths of GSCM during 300–600 h of aging, the transverse tension strengths of the rest of the materials were less than the longitudinal ones.

The ROTS describes the deviation of the tension strength in longitudinal and transverse directions; the biaxial tensile strength is consistent if the ROTS value equals one, and the material can be regarded as isotropic. Among all the geonet materials, the variation in the ROTS of the SG with aging-elapsed time was the most significant, and the curve showed a double-peak feature. However, the rest of the variation curves only exhibited a single-peak feature, and the peak value of the ROTS appeared by the time of 400 h. Moreover, the fluctuation of the ROTS curve of the HPG was the smallest, and the ROTS only varied within 0.1. Conversely, the variation in the SG and the GG curves was quite strong; their deviation from the ROTS was more than 0.35.

**Figure 5:**
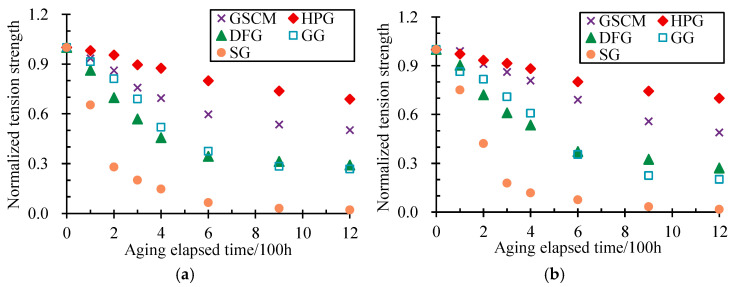
Variation in longitudinal and transverse normalized tension strength with aging-elapsed time. (**a**) Longitudinal. (**b**) Transverse.

To further analyze the difference in tensile strength reduction among the geonet materials during aging, the results of tension strength after different aging-elapsed times were normalized by dividing them into the initial tension strength. Figure 5 depicts the longitudinal and transverse normalized tension strength variation with the aging-elapsed time of all geonet materials.

Figure 5 shows that the normalized tension strength of all geonet materials generally decreased with the growth of the aging-elapsed time. Moreover, the aging-elapsed time of 600 h was likely to be a boundary point; the strength underwent a considerable reduction when the aging-elapsed time was less than 600 h, while the variations gradually tended to be stable when beyond 600 h. This phenomenon occurred in all geonet materials, in longitudinal and transverse directions. However, the content of their strength loss was quite different. Ranging from large to small, the order of the normalized tension strength after 600 h of aging was HPG (0.80, 0.80), GSCM (0.60, 0.69), GG (0.38, 0.35), DFG (0.34, 0.37), and SG (0.06, 0.08), respectively. The first value in the bracket is the normalized tension strength in the longitudinal direction; otherwise, it is the one in the transverse direction. By the time aging increased by 1200 h, there was a slight change in the order to HPG (0.69, 0.70), GSCM (0.50, 0.49), DFG (0.29, 0.27), GG (0.27, 0.20), and SG (0.02, 0.02), respectively. It can be seen that the HPG and the GSCM have better anti-aging characteristics compared to the other testing materials, whose loss of tension strength after 1200 h of aging was approximately 30% and 50%, respectively. At the same time, the DFG and the GG had lost their tension strength by more than 70%, and the worst SG almost lost its strength.

#### 3.1.2. Variation in Percentage of Breaking Elongation (PBE)

**Figure 6:**
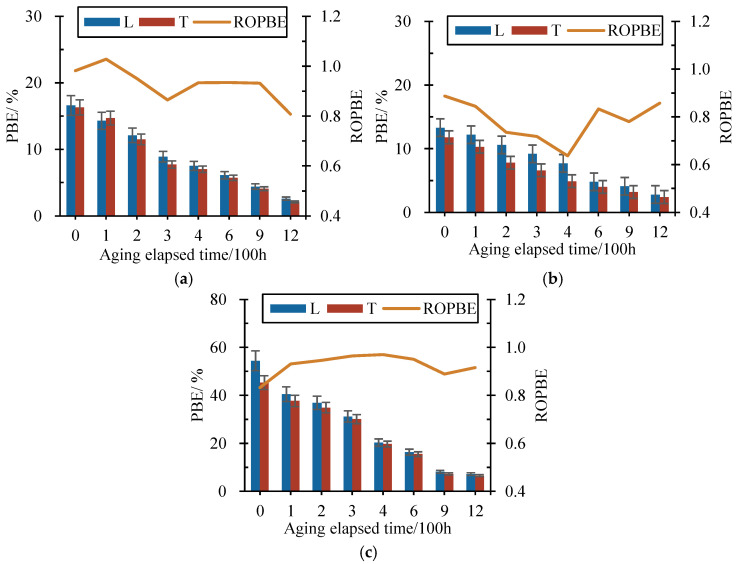

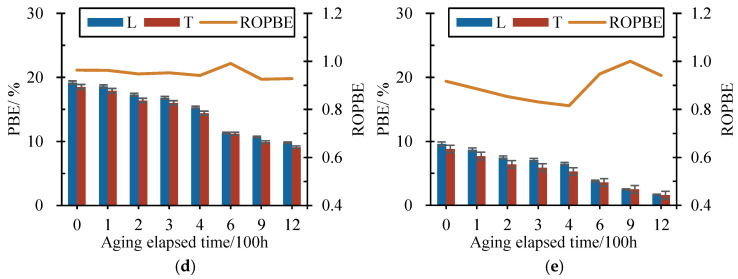
Variation in longitudinal (L) and transverse (T) PBE and ratio of orthogonal PBE (ROPBE) with aging-elapsed time. (**a**) SG. (**b**) GG. (**c**) DFG. (**d**) HPG. (**e**) GSCM.

When a material is subjected to the external load until it breaks, the ratio of the elongation length after tension to the length before tension is called the percentage of breaking elongation (PBE), which indicates the elongation capacity of the material under the maximum external load. Figure 6 demonstrates the variation in longitudinal (L) and transverse (T) PBE of each type of the 3D geonets with aging-elapsed time, where the ratios of orthogonal PBE (ROPBE) are also depicted calculated by dividing the transverse PBEs by longitudinal ones.

As shown in Figure 6, with the increase in the aging time, the longitudinal and transverse PBEs of all types of geonet materials showed a continuous decline in trend. Unlike the tension strength, the GSCM had the smallest PBE, which was less than 10% before aging and was reduced by less than 2% after 1200 h of aging. The magnitude of PBE of the DFG was significant; its initial PBE was the largest, approximately 5–6 times that of the GSCM, but it also decreased to 7.1% in longitudinal and 6.5% in transverse after 1200 h of aging, which were less than that of the HPG at that time. The reduction in the PBE of the SG and the GG was at the medium level among all the materials; the PBE of the SG decreased from 16.6% to 2.6% in longitudinal and 16.3% to 2.1% in transverse, while the PBE of the GG decreased from 13.3% to 2.8% in longitudinal and 11.8% to 2.4% in transverse. In addition, the transverse PBEs of all the geonet materials were less than the longitudinal ones.

Similar to the ROTS, the ROPBE also indicated the difference in the PBE between the orthogonal directions. It was observed that the ROPBE of the HPG also varied less than 0.1 with the aging-elapsed time; this trend was consistent with the variation in ROTS. The ROPBE curves of the SG and the GG exhibited an ‘M’-shaped double-peaked distribution, and their variation was the most significant among all the materials, where the variation in the ROPBE was beyond 0.2. The ROPBE curves of the DFG and the GSCM showed an ‘S’ shaped single-peaked distribution, the ROPBE of which varied in the range of 0.1–0.2 with the aging-elapsed time.

**Figure 7:**
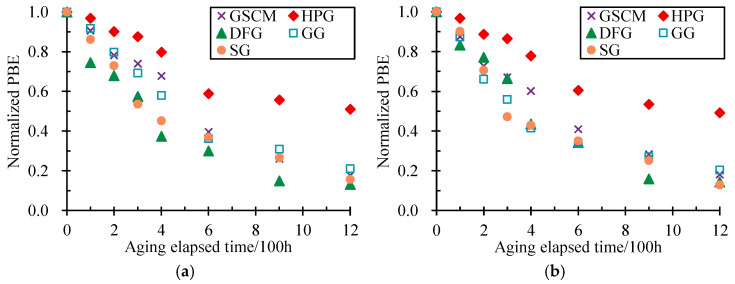
Variation in longitudinal and transverse normalized PBE with aging-elapsed time. (**a**) Longitudinal. (**b**) Transverse.

For the same reason, the PBE results after different aging-elapsed times were normalized by dividing them into the initial PBE. Figure 7 depicts the variation in longitudinal and transverse normalized PBE with the aging-elapsed time of all types of geonet materials.

As depicted in Figure 7, it was found that the longitudinal and transverse normalized PBE all firstly decreased with the aging-elapsed time and then tended to be stable beyond 600 h. After 600 h of aging, the longitudinal normalized PBEs of SG, GG, DFG, HPG, and GSCM were, respectively, reduced to 0.37, 0.36, 0.30, 0.59, and 0.40, while the transverse normalized PBEs were 0.35, 0.34, 0.34, 0.61, and 0.41, respectively. Compared to the initial PBE before aging, the PBE of the HPG decreased by approximately 40%, while the remaining materials decreased their PBE by 60–70%. With the increase in aging-elapsed time, after 1200 h of aging, the longitudinal normalized PBEs were 0.16, 0.21, 0.16, 0.51, and 0.18, respectively, and the transverse normalized PBEs were 0.13, 0.20, 0.14, 0.49, and 0.18. At that time, the PBE of the HPG was reduced by approximately 50%, while the rest of the materials were reduced by 79–87% compared to their initial PBE. It suggests that the aging process significantly reduces the PBE of the geonet materials, which would become more brittle after aging. The high temperature during aging promoted the chemical reactions within the material that increased the crystallinity and led to the breakage of molecular chains, thus reducing the material’s flexibility.

#### 3.1.3. Variation in Peer Strength

**Figure 8:**
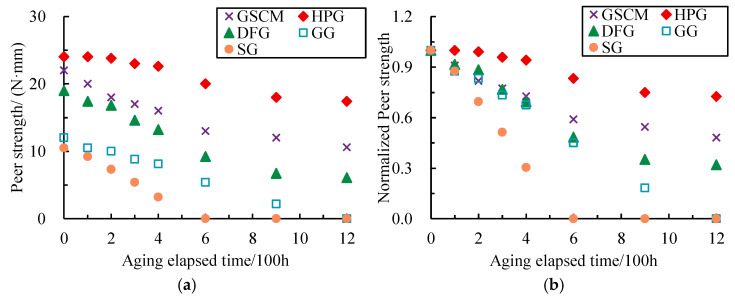
Variation in peer strength and normalized peer strength with aging-elapsed time. (**a**) Peer strength. (**b**) Normalized peer strength.

Peel strength is the maximum force required to peel a material bonded together from a contact surface per unit width. Figure 8 depicts the variation in peer strength and the normalized peer strength with aging-elapsed time. The normalized peer strength was calculated by dividing it into the initial peer strength before aging.

As shown in Figure 10, the peer strength linearly decreased with the aging-elapsed time before 600 h of aging; then, its decreased tendency slowed down and gradually reached a stable stage at up to 1200 h of aging. The SG totally lost its peer strength after 600 h of aging, and the GG completely lost this property after 1200 h of aging. The rest of the materials retained some peer strength, with the DFG at 0.32 times of initial peer strength, the HPG at 0.73, and the GSCM at 0.48.

It indicated that the aging process significantly negatively impacted the stability of geonet interlayer connections, especially for those materials bonded together by spot welding or thermal bonding (e.g., the SG and the GG). The bonded interlayer part of the 3D geonets would first melt under high temperature and continuous light, leading to an interlayer separation of their composite multilayer structure.

### 3.2. Aging Effect on Micro-Surface Morphology

**Figure 9:**
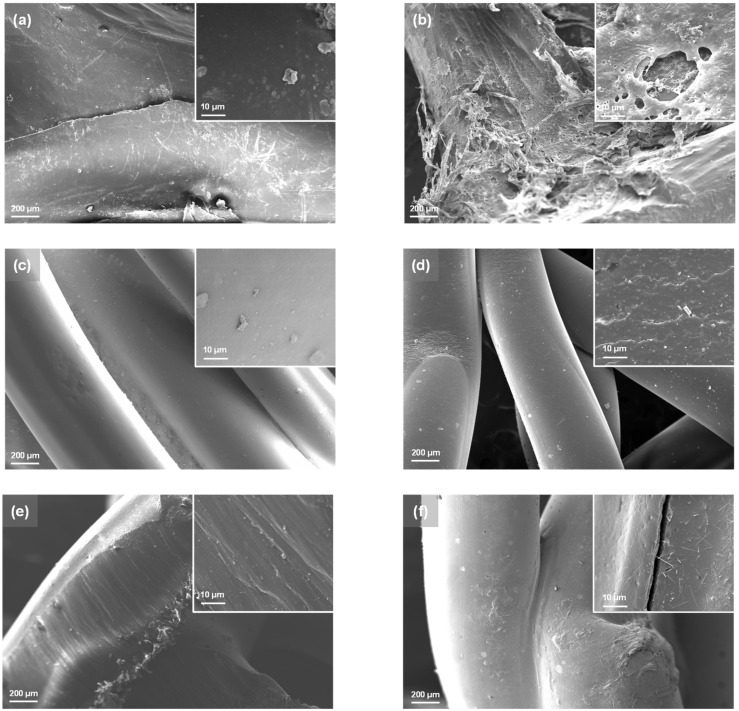
SEM images of PE geonet (**a**,**b**), PP geonet (**c**,**d**), and PA geonet (**e**,**f**), respectively.

Using a scanning electron microscope (SEM), the micro-surface morphology of the SG (made of PE), HPG (made of PP), and GSCM (made of PA) before and after 1200 h of aging was observed. Figure 9 depicts the micro-surface morphology of the geonets material, with the first column showing the SEM photos of the materials before aging.

The surfaces of all three materials were relatively smooth before aging, especially the PP geonet, which appeared to be very smooth and uniform (Figure 9c). Meanwhile, the PE geonet showed a few scratches on the surface (Figure 9a), and the PA geonet presented a parallel distribution of textures (Figure 9e). Moreover, all the geonets had some raised particles on the part of the material surface. Significant changes were generated on the geonet surface after 1200 h of the aging process. As shown in Figure 9b, a curly flocculent structure appeared on the surface of the PE geonet, while part of the surface melted and cavities appeared. The PP and PA geonets were much better and still exhibited smooth inherent fiber surfaces. However, folds and crumbs were also observed in the PP geonet from Figure 9d. As for the PA geonet (Figure 9f), its surface became much rougher, the bumps on the surface were increased, and cracks appeared with needle-like fibers distributed around.

From the SEM observation, it can be concluded that the difference in texture of the material will be reflected in its aging process. Furthermore, the material with a smooth surface showed a strong ability against physical properties during aging. The results were consistent with those of the tension strength test and peer strength test, where the PP geonet (HPG) had the strongest ability against aging and the smoothest surface.

### 3.3. Prediction on Service Life of Geonet Materials

Ultraviolet (UV) irradiation is the most critical factor that triggers photo-oxidative aging of materials. Radiant energy can represent the amount of UV irradiation and is numerically equal to the product of radiation intensity and radiation duration. Based on the irradiation intensity and radiant energy of artificial accelerated aging and natural atmospheric exposure aging, the equivalent conversion rate between the two aging conditions can be calculated according to Equation (1), and then the aging-elapsed time under actual natural conditions can be simulated by the laboratory artificial accelerated aging test:

(1) $$A_{u}=\frac{Q_{r}}{Q_{z}}$$

where *Q_r_* is the cumulative radiation energy in the AAAT testing equipment within a specified time, J/cm^2^; *Q_z_* is the radiation energy of solar UV under actual natural condition within the same time, J/cm^2^; and *A_u_* is the equivalent conversion rate.

According to the Chinese classification of solar energy resources: global radiation GB/T 31155 (China Meteorological Administration 2014) [32], Hunan Province is in Zone IV of solar radiation intensity where the total solar radiation and light duration are, respectively, 4200~5000 MJ/m^2^ and 1400~2000 h per year. Considering that UV radiation accounts for 5% of the total solar energy [33], the annual UV radiation energy was calculated to be 210~250 MJ/m^2^. Accordingly, the annual hourly UV radiation energy was about 2.40~2.85 kJ/m^2^.

As mentioned in the test procedure and methods, the light intensity was set to 600 W/m^2^ during the accelerated aging period, and the cycle process was 11 h of light and 1 h of condensation. The radiation energy and the equivalent conversion rate were calculated; the results are shown in Table 2.

Table 2 depicts that the equivalent conversion rate varies from 58 to 69 depending on the annual hourly UV radiation energy, which was about 2.40~2.85 kJ/m^2^. Therefore, the aging-elapsed time under actual atmospheric exposure conditions should also be 58~69 times that in the AAAT; it is equivalent to that in the natural environment of Hunan Province; 1200 h of AAAT corresponds to 7.28~8.66 years of atmospheric exposure time.

**Figure 10:**
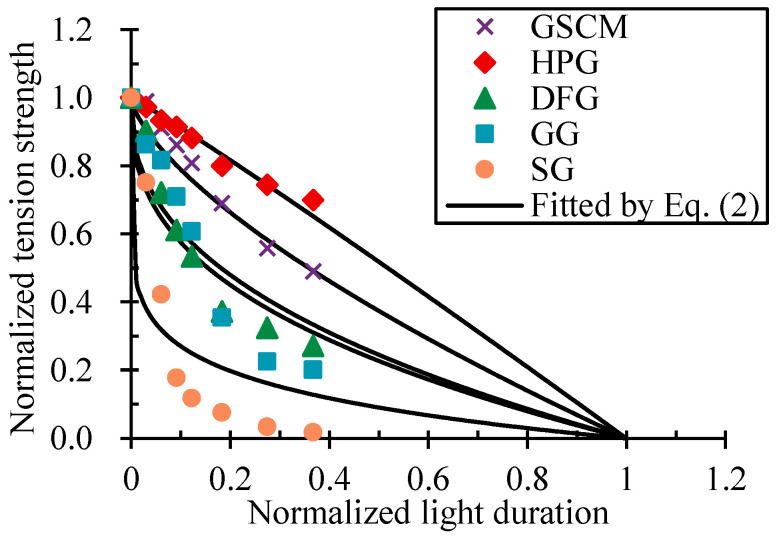
Variation in normalized tension strength with normalized light duration.

Considering that the minimum service life of the geonet used in slope protection is 20 years, the light duration in Table 2 was normalized by dividing it by 175,200 h (20 years). Based on the results shown in Figure 5, the variations in normalized tension strength with normalized light duration among these materials are depicted in Figure 10, where the equivalent conversion rate was set as 58.

The nonlinear variation in these scatters was observed in each test series of Figure 10, where the relationship between the normalized tension strength and light duration can be described by Equation (2):

(2) $$\frac{\tau }{\tau_{initial}}=1-{\left(\frac{T}{T_{d,\min}}\right)}^{n}$$

where *τ* is the tension strength of geonet material during service life, kN/m; *τ_initial_* is the initial tension strength of geonet material before aging, kN/m; *T* is the light duration, which equals the aging-elapsed time in AAAT multiplying the equivalent conversion rate; *T_d_*_,min_ is the minimum designed service life, which is set as 20 years in this study.

The values of the fitting parameters and correlation coefficient are depicted in Table 3. The fitting results showed a good correlation of the DFG, HPG, and GSCM, whose correlation coefficients *R*^2^ were all above 0.92. The fitting results of SG and GG were relatively worse, 0.8063 and 0.8749, respectively. The results demonstrate the effectiveness of the proposed prediction in capturing the degradation of tension behavior under actual solar radiation. With further refinement, the approach holds promise as a practical tool for facilitating the wider usage of geonet materials in geotechnical applications.

## 4. Conclusions

This study conducted a comprehensive experimental investigation into the strength degradation of geonets against ultraviolet radiation and developed predictive methodologies to estimate tension strengths under actual atmospheric exposure conditions. Based on the results and findings, the following conclusions can be drawn:

(1) The tension strength of different geonet materials and directions all showed a huge descending trend, with the aging-elapsed time increasing to 600 h. The high-performance geonet (HPG) and the gridded soil conservation mat (GSCM) have better anti-aging characteristics compared to the rest of the materials, with less loss of tension strength after 1200 h of aging.
(2) The percentage of breaking elongation (PBE) showed a continuous decline in trend with the increase in aging-elapsed time. Compared to the initial PBE before aging, the PBE of the HPG decreased by approximately 50% after 1200 h of aging, while the rest of the materials decreased their PBE by 79–87%.
(3) The SG fully lost its peer strength after 600 h of aging, and the GG also completely lost it after 1200 h of aging, which indicated that the aging process had a significantly negative impact on the stability of geonet interlayer connections, especially for those materials bonded together by spot welding or thermal bonding.
(4) Significant changes were generated on the geonet surface after 1200 h of aging. A curly flocculent structure appeared on the surface of the PE geonet, while part of the surface melted, and cavities appeared. The PP and PA geonets were much better and still exhibited smooth inherent fiber surfaces. Materials with smooth surfaces showed strong ability against physical properties during aging.
(5) A simple predictive equation was developed to estimate the tension strength of geonet materials from natural atmospheric exposure conditions via normalization. Fitting results affirmed the predictive technique as a useful engineering tool for tension strength assessments, with all correlation coefficients above 0.80.

In conclusion, these findings provide an improved understanding of more realistic characterization and utilization of geonets in geotechnical applications. However, the laboratory-accelerated aging tests need more extensive time periods for long-term service life prediction. The predictive technique should further be modified by considering the reduction in solar-radiation-induced by vegetation, and it should also be further validated by numerical simulations and in situ tests under natural atmospheric exposure conditions.

## Figures and Tables

**Table 1:** The basic properties of the slope-protection materials.

Type	Unit Mass per Area/(g·m^2^)	Scale of Single Mesh/mm	Thickness/mm
SG	243	(6 × 8) ± 1	≥12
GG	447	(10 × 10) ± 1	≥14
DFG	534	(10 × 10) ± 1	≥16
HPG	750	(10 × 10) ± 1	≥23
GSCM	793	(10 × 15) ± 1	≥9

**Table 2:** Results of radiation energy and equivalent conversion rate.

Aging elapsed time (h)	0	100	200	300	400	600	900	1200
Light duration (h)	0	92	184	275	367	550	825	1100
Energy in AAAT (MJ/m^2^)	0	15.3	30.7	45.8	61.2	91.7	137.5	183.3
Energy of solar UV (MJ/m^2^)	0	0.2~0.3	0.4~0.5	0.7~0.8	0.9~1.0	1.3~1.6	2.0~2.4	2.6~3.1
Equivalent conversion rate	58~69							

**Table 3:** Results of fitting parameter and correlation coefficient.

Type of Material	SG	GG	DFG	HPG	GSCM
Fitting parameter (*n*)	0.1367	0.4062	0.3720	1.0537	0.6762
correlation coefficient (*R*^2^)	0.8063	0.8749	0.9237	0.9923	0.9679

## Data Availability

The raw data supporting the conclusions of this article will be made available by the authors on request.
